# Involvement of Hsp90 and cyclophilins in intoxication by AIP56, a metalloprotease toxin from *Photobacterium damselae* subsp. *piscicida*

**DOI:** 10.1038/s41598-019-45240-w

**Published:** 2019-06-21

**Authors:** Inês S. Rodrigues, Liliana M. G. Pereira, Johnny Lisboa, Cassilda Pereira, Pedro Oliveira, Nuno M. S. dos Santos, Ana do Vale

**Affiliations:** 1 Fish Immunology and Vaccinology Group, IBMC-Instituto de Biologia Molecular e Celular, Universidade do Porto, Porto, Portugal; 2 i3S-Instituto de Investigação e Inovação em Saúde, Universidade do Porto, Porto, Portugal; 3 EPIUnit, ICBAS-Instituto de Ciências Biomédicas Abel Salazar, Universidade do Porto, Porto, Portugal

**Keywords:** Cellular microbiology, Bacterial toxins

## Abstract

AIP56 (apoptosis inducing protein of 56 kDa) is a key virulence factor secreted by virulent strains of *Photobacterium damselae* subsp. *piscicida* (*Phdp*), a Gram-negative bacterium that causes septicemic infections in several warm water marine fish species. AIP56 is systemically disseminated during infection and induces massive apoptosis of host macrophages and neutrophils, playing a decisive role in the disease outcome. AIP56 is a single-chain AB-type toxin, being composed by a metalloprotease A domain located at the N-terminal region connected to a C-terminal B domain, required for internalization of the toxin into susceptible cells. After binding to a still unidentified surface receptor, AIP56 is internalised through clathrin-mediated endocytosis, reaches early endosomes and translocates into the cytosol through a mechanism requiring endosomal acidification and involving low pH-induced unfolding of the toxin. At the cytosol, the catalytic domain of AIP56 cleaves NF-κB p65, leading to the apoptotic death of the intoxicated cells. It has been reported that host cytosolic factors, including host cell chaperones such as heat shock protein 90 (Hsp90) and peptidyl-prolyl *cis/trans* isomerases (PPIases), namely cyclophilin A/D (Cyp) and FK506-binding proteins (FKBP) are involved in the uptake of several bacterial AB toxins with ADP-ribosylating activity, but are dispensable for the uptake of other AB toxins with different enzymatic activities, such as *Bacillus anthracis* lethal toxin (a metalloprotease) or the large glycosylating toxins A and B of *Clostridium difficile*. Based on these findings, it has been proposed that the requirement for Hsp90/PPIases is a common and specific characteristic of ADP-ribosylating toxins. In the present work, we demonstrate that Hsp90 and the PPIases cyclophilin A/D are required for efficient intoxication by the metalloprotease toxin AIP56. We further show that those host cell factors interact with AIP56 *in vitro* and that the interactions increase when AIP56 is unfolded. The interaction with Hsp90 was also demonstrated in intact cells, at 30 min post-treatment with AIP56, suggesting that it occurs during or shortly after translocation of the toxin from endosomes into the cytosol. Based on these findings, we propose that the participation of Hsp90 and Cyp in bacterial toxin entry may be more disseminated than initially expected, and may include toxins with different catalytic activities.

## Introduction

AIP56 (**A**poptosis **I**nducing **P**rotein of 56 kDa) is a plasmid-encoded toxin produced by virulent strains of *Photobacterium damselae* subsp. *piscicida* (*Phdp*), a Gram-negative pathogen that infects and causes high mortalities in several warm water fish species worldwide, frequently leading to serious economic losses in aquaculture farms^1^. AIP56 is the main virulence factor of *Phdp*, playing a decisive role in the establishment of *Phdp* infections, which are characterised by the occurrence of generalized bacteraemia and extensive cytopathology with abundant tissue necrosis^2^. The toxin is a type II secreted effector^3^ abundantly secreted *in vitro*^1^ and *in vivo*^2^. In *Phdp* infections, secreted AIP56 disseminates systemically and induces apoptosis of host macrophages and neutrophils^2^. The destruction of these main players responsible for the phagocytic defence contributes to the severity of *Phdp* infections by facilitating the survival and extensive extracellular multiplication of the pathogen^2^. Concurrently, the destruction of macrophages, which are important for the elimination of apoptotic cells, leads to an inefficient clearance of apoptotic phagocytes and culminates with their lysis by secondary necrosis, with release of their cytotoxic intracellular contents that cause tissue injury and contribute to the genesis of the characteristic cytopathology of *Phdp*^2,4,5^.

AIP56 is a single-chain AB-type toxin^6^. It is composed by a metalloprotease A domain located at the N-terminal region connected to a C-terminal B domain, involved in the binding and internalization of the toxin into susceptible cells^6^. AIP56 is a so-called “short-trip” toxin that reaches its intracellular target using a mechanism that is conserved between fish and mammalian cells^7^. After binding to a still unidentified surface receptor of susceptible cells, AIP56 is internalised through clathrin-mediated endocytosis, reaches early endosomes and translocates into the cytosol through a mechanism requiring endosomal acidification and involving low pH-induced unfolding of the toxin^7^. At the cytosol, the catalytic domain of AIP56 cleaves an evolutionarily conserved peptide bond of the Rel homology domain of NF-κB p65, leading to the removal of residues required for p65-DNA interaction and compromising NF-κB activity^6^. This activity of AIP56 causes a complete depletion of p65 and culminates with the death of the intoxicated cells by apoptosis^6,7^ through a pathway involving activation of caspases-8, -9 and -3, loss of mitochondrial membrane potential, translocation of cytochrome c to the cytosol and hyperproduction of ROS^2,8–11^.

The requirement of host cell cytosolic factors for the entry process of a bacterial toxin was first reported for diphtheria toxin^12,13^. Following these initial observations, heat shock protein 90 (Hsp90) has been identified as a host cell cytosolic factor essential for efficient intoxication by the ADP-ribosylating toxins diphtheria toxin^14^, *Clostridium botulinum* C2 toxin^15^, *C. perfringens* iota-toxin and *C. difficile* ADP-ribosyltransferase^16^. In the last decade, several reports confirmed the involvement of Hsp90 in membrane translocation of other ADP-ribosylating toxins and showed that peptidyl-prolyl cis/trans isomerases (PPIases) of the cyclophilin (Cyp) and FK506-binding (FKBP) families act in concert with Hsp90 in assisting the translocation process^17–23^. In contrast, Hsp90 and PPIases were found to be dispensable for the uptake of other AB toxins with different enzymatic activities, such as the metalloprotease lethal toxin from *Bacillus anthracis*^24,25^ and the large glycosylating toxins A and B of *Clostridium difficile*^15,17–19^, leading to the hypothesis that the requirement for Hsp90/PPIases is a common and specific characteristic of only ADP-ribosylating toxins^22^.

In the present work, we demonstrate that Hsp90 and the PPIases cyclophilin A/D are required for efficient intoxication by the metalloprotease toxin AIP56. We further show that those host cell factors interact with AIP56 *in vitro* and that the interactions are enhanced if AIP56 is unfolded. The interaction with Hsp90 was also demonstrated in intact cells, at 30 min post-treatment with AIP56, suggesting that it occurs during or shortly after translocation of the toxin from endosomes into the cytosol. Altogether, these results suggest that Hsp90/cyclophilins facilitate AIP56 intoxication by assisting its translocation and/or by promoting the regain of a folded, active state of the toxin at the cytosol.

## Results

### Pharmacological inhibition of host cell Hsp90 or cyclophilins inhibits intoxication of mBMDM by AIP56

**Figure 1:**
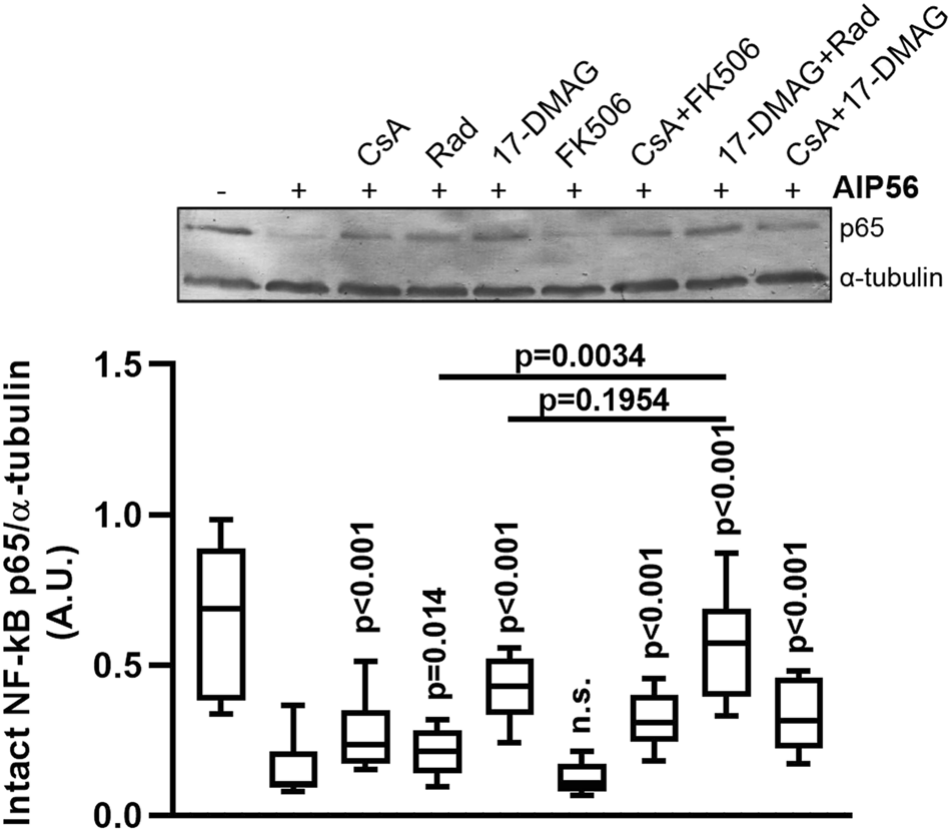
Pharmacological inhibition of Hsp90 and cyclophilins inhibits intoxication of mBMDM with AIP56. mBMDM were pre-treated with the indicated inhibitors (10 µM Rad and 20 µM 17-DMAG, CspA or FK506), alone or at the indicated combinations, prior to intoxication with 170 nM AIP56 for 30 min on ice plus 15 min at 37 °C. The medium was replaced by fresh medium with inhibitor(s), and after 2 h at 37 °C, cleavage of p65 was assessed by western blotting (chromogenic detection). A representative blot is shown (the full-length blot is presented in Fig. S7). The box-plot shows the quantification of the blots (n = 6 independent experiments). Loading correction was achieved by dividing the density of p65 by the respective density of α-tubulin. Statistical significance was tested by one-way ANOVA and p-values for individual comparisons to cells treated only with toxin were calculated using the Dunnett’s test.

To investigate whether the activity of the host cell chaperone Hsp90 and PPIases, namely cyclophilins (Cyps) and FK506-binding proteins (FKBPs), are involved in the cellular uptake of the AIP56 toxin in mouse bonemarrow derived macrophages (mBMDM), we pre-incubated mBMDM with specific pharmacological inhibitors before intoxication with AIP56 and assessed intoxication by quantifying the AIP56-induced NF-κB p65 cleavage. The choice of this readout for monitoring intoxication was based on the fact that it is an early and specific indicator to detect arrival of AIP56 into the cytosol and, therefore, is not influenced by other factors (i.e., under our experimental conditions, the processing of p65 is only related to the catalytic activity of AIP56). Several pharmacological inhibitors specific for Hsp90 and PPIases are commercially available and have been successfully used to investigate the involvement of those host cell factors in the uptake of different bacterial toxins in mammalian cells^15,18,20^. Amongst those inhibitors are the cyclosporine A (CsA) which specifically targets cyclophilins^26^, FK506 which specifically inhibits FKBPs^27^ and radicicol (Rad) and 17-DMAG, both targeting Hsp90. Although structurally different, both Rad and 17-DMAG block the activity of Hsp90 by binding with high affinity to the ATPase-binding pocket of the chaperone, although to different sites^28–30^. The concentrations of the inhibitors to be used were determined based on previous studies with ADP-ribosylating toxins reported in the literature^19–21,31^ and in preliminary toxicity tests in mBMDM (see Supplementary Fig. S1). A final concentration of 20 μM was used for all inhibitors except Rad, because in this case, concentrations above 10 μM were toxic for mBMDM. To validate the function of the specific inhibitors Rad, 17-DMAG, CsA and FK506, at the selected concentrations, in mBMDM, we took advantage of the His-tagged ADP-ribosyltransferase domain hvr of TccC3 (His-TccC3) that was shown to enter cells through the anthrax protective antigen (PA) pore in a Hsp90, cyclophilin A and FKBPs-dependent way^20^. mBMDM were pre-treated with the inhibitors for 1 h prior to the addition of PA + His-TccC3 and intoxication evaluated by quantifying the percentage of rounded cells, as described^20^. Pre-treatment of cells with Rad, 17-DMAG, CsA or FK506 significantly inhibited intoxication (Supplementary Fig. S2), confirming the activity of the inhibitors in our experimental conditions. To address if Hsp90 and PPIases are involved in AIP56 intoxication, mBMDM were left untreated or pre-treated for 1 h with Rad, 17-DMAG, CsA and FK506, alone or combined, prior intoxication with AIP56, and intoxication determined by analysing AIP56-dependent p65 cleavage by western blotting^6,7^. At the applied concentrations and under the experimental conditions used, treatment with the inhibitors alone had no effect on NF-κB p65 levels (Supplementary Fig. S3) and none of the vehicles used to dissolve the inhibitors affected AIP56-dependent p65 cleavage (Supplementary Fig. S4). As shown in Fig. 1, AIP56-dependent p65 cleavage was not affected by pre-treatment of mBMDM with FK506. In contrast, a significant inhibition of the p65 cleavage was observed when cells were pre-treated with CsA, Rad or 17-DMAG before incubation with AIP56 (Fig. 1). Furthermore, Rad and 17-DMAG act cumulatively, since when Rad was applied in combination with 17-DMAG, the inhibitory effect was stronger when compared to pre-treatment with Rad (p = 0.0034) or 17-DMAG alone (although in this case, the difference did not reach statistical significance; p = 0.1954), resulting in an almost complete inhibition of p65 cleavage (Fig. 1). The inhibitory effect of CsA in AIP56 intoxication was slight when compared to the inhibition observed in experiments with ADP-ribosylating toxins, such as TccC3 (see Fig. S2). This may be due to specificities of the AIP56 intoxication mechanism that make the toxin less dependent on CsA than the ADP-ribosylating toxins. Taken together, these results indicate that although FKBPs are dispensable for AIP56 intoxication, Hsp90 and cyclophilins are involved in AIP56 toxicity.

### Cyclosporin A, 17-DMAG and radicicol do not inhibit the metalloprotease activity of AIP56 neither AIP56 endocytosis

**Figure 2:**
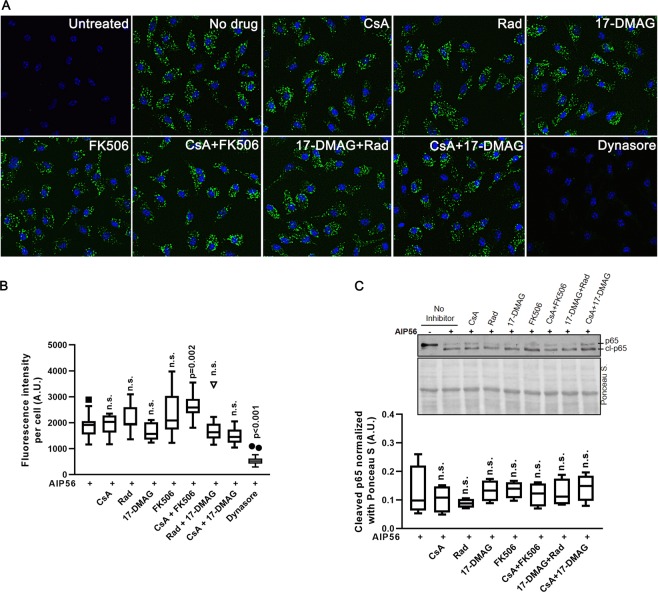
Pharmacological inhibition of Hsp90 and cyclophilins has no effect on the endocytic uptake and catalytic activity of AIP56. (**A**) mBMDM were left untreated or treated with the indicated inhibitors (10 µM Rad and 20 µM 17-DMAG, CspA or FK506), alone or at the indicated combinations, before incubation with 170 nM AIP56-488 for 30 min on ice followed by 15 min at 37 °C. As control, cells were incubated with 80 µM of the dynamin inhibitor dynasore, previously shown to inhibit AIP56 endocytosis. The cells were fixed and nuclei were stained with DAPI. Images were acquired using IN Cell Analyzer 2000 (GE Healthcare) with a 20 × objective. Images shown are representative microscopic fields from one experiment. (**B**) Box-plot showing the quantification of the fluorescence intensity per cell (n = 3 independent experiments). In each experiment, a minimum of 500 cells per condition were analysed. Statistical significance was tested by one-way ANOVA and p-values for individual comparisons to cells treated only with toxin were calculated using the Dunnett’s test. (**C**) The metalloprotease activity of AIP56 is not affected by the inhibitors used in this study. Sea bass peritoneal cell lysates (containing sea bass p65) were incubated for 3 h at 22 °C with 100 nM recombinant AIP56 in the presence of Rad, CsA, 17-DMAG or FK506, alone or combined, and cleavage of p65 assessed by western blotting (chromogenic detection). Lysates incubated with the toxin in the absence of the inhibitors were used as controls. A representative blot is shown (the full-length blot is presented in Fig. S7). The box-plot shows the quantification of the cleaved p65 (n = 4 independent experiments). Loading correction was achieved by dividing the density of the cleaved p65 by the respective density of Ponceau S. Statistical significance was tested by one-way ANOVA and p-values for individual comparisons to cell lysates treated only with toxin were calculated using the Dunnett’s test.

The protection of mouse macrophages from intoxication by AIP56 after pharmacological inhibition of Hsp90 and cyclophilins (Fig. 1) does not provide information about the intoxication step that is inhibited. To understand better the role of Hsp90 and cyclophilins in AIP56 intoxication, we analysed the effect of the pharmacological inhibition of those host cell proteins in different phases of the AIP56 uptake. First, we evaluated if the inhibitors affected the endocytic uptake of AIP56, by performing an endocytosis experiment (Fig. 2A). Cells were left untreated or treated for 1 h with CsA, Rad, 17-DMAG or FK506, alone or combined, prior to incubation with AIP56 labelled with Alexa Fluor 488 (AIP56-488) for 30 min on ice to allow binding of the toxin to the cells plus 15 min at 37 °C to allow toxin’s endocytosis. As control, cells were pre-treated with dynasore, a dynamin inhibitor previously shown to inhibit AIP56 endocytosis^7^. Subsequently, cells were washed, fixed and the endocytosed toxin analysed by fluorescence microscopy. As shown in Fig. 2A,B, apart from dynasore, none of the inhibitors/inhibitors’ combinations inhibited the initial endocytic uptake of the toxin. Next, we tested if the inhibitors affect the AIP56 metalloprotease activity. For this, we used an *in vitro* assay in which recombinant AIP56 was incubated with sea bass cell lysates, which contain p65, in the presence or absence of the inhibitors, followed by detection of p65 cleavage by western blotting. As shown in Fig. 2C, none of the inhibitors used impaired the AIP56 proteolytic activity. Altogether, these findings show that the observed inhibition of AIP56 intoxication was not due to direct impairment of enzymatic activity caused by the treatments, neither deficient endocytosis of the toxin, suggesting that Hsp90 and cyclophilins are required for later steps of the intoxication process, i.e., in assistance of AIP56 translocation and or refolding at the cytosol.

### Hsp90 and cyclophilins interact with AIP56 *in vitro*

**Figure 3:**
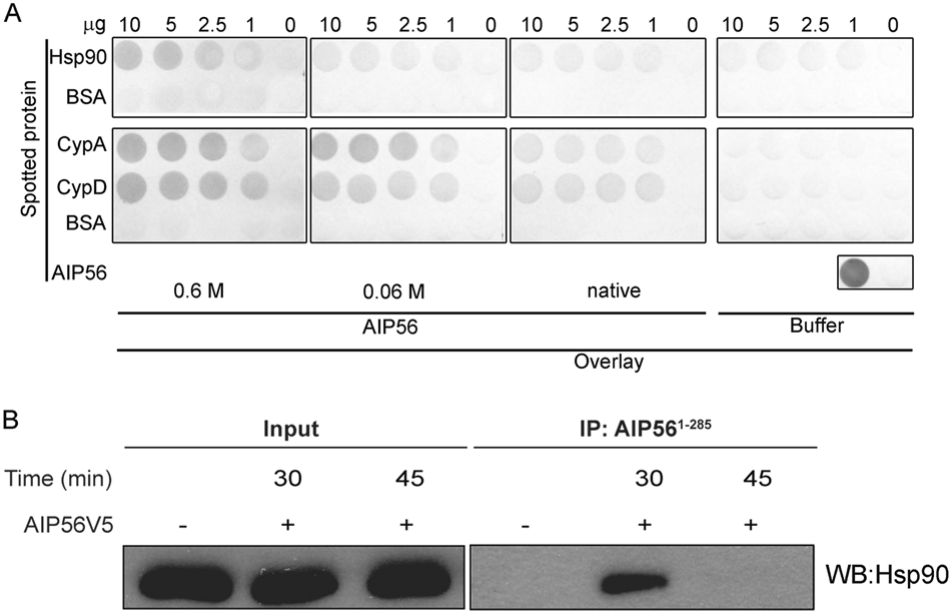
Interaction of AIP56 with Hsp90 and cyclophilins *in vitro* and during mBMDM intoxication. (**A**) Dot blot analysis of the interaction between AIP56 and Hsp90 or cyclophilins A/D. Nitrocellulose membranes spotted with 10, 5, 2.5 or 1 µg of Hsp90, CypA or CypD were incubated with 170 nM native AIP56 or AIP56 that was denatured by incubation in 6 M guanidine hydrochloride (GuHCl) for 1 h at room temperature and then diluted to achieve a final concentration of 0.6 M or 0.06 M GuHCl, respectively. As control, equivalent membranes were incubated with buffer without toxin. The interaction of AIP56 with the different immobilized proteins was detected with a mixture of anti-AIP56^1–285^ and anti-AIP56^286–497^ rabbit polyclonal antibodies followed by a phosphatase alkaline-conjugated secondary antibody (chromogenic detection). The dot-blot was repeated four times. The blots shown were originally from the same membrane that was cropped to allow separate incubations with the different overlay mixtures. After these incubations, and in all the subsequent steps, the membranes spotted with Hsp90 or CypA/D were grouped and each group was processed together in the same container. The Ponceau S staining of the membranes is shown in Supplementary Fig. S6 (**B**) Analysis of the co-precipitation of Hsp90 with AIP56 in lysates from intoxicated cells. Cells were left untreated (control) or incubated with 285 nM AIP56V5 for the indicated times. Cytosolic fractions were obtained and incubated with anti-AIP56^1–285^ coated beads overnight at 4 °C. The presence of co-precipitated Hsp90 was analysed by western blotting with an anti-Hsp90 antibody, followed by a peroxidase-linked secondary antibody (ECL detection). Input fractions taken from the respective samples prior to bead incubation were run as controls. All samples shown were from the same experiment; IPs and input samples were analysed in two different gels. Full-length blots superimposed into the Ponceau S scans are presented in Fig. S7.

Based on the hypothesis that Hsp90 and cyclophilins are involved in AIP56 intoxication by facilitating the translocation and/or refolding of the toxin at the cytosol, we performed dot-blot experiments to determine if AIP56 interacts with those host cell factors *in vitro*. Host proteins tested included Hsp90, cyclophilin A (CypA), the most prominent cyclophilin in mammalian cells and the major target for CsA^32^ as well as cyclophilin D (CypD), which is also inhibited by CsA. Thus, different doses of recombinant Hsp90, CypA or CypD were loaded in nitrocellulose membranes which were then incubated with native AIP56 or AIP56 previously denatured by incubation in 6 M guanidine hydrochloride (GuHCl) and then diluted to achieve final concentrations of 0.6 M or 0.06 M GuHCl (the unfolding effect of GuHCl is shown in Supplementary Fig. S5). Membranes incubated with buffer without toxin were used as controls and bound AIP56 was detected using a mixture of anti-AIP56^1–285^ and anti-AIP56^286–497^ antibodies. As shown in Fig. 3A, a slight interaction of native AIP56 with CypA and D was observed. A similar binding pattern was obtained when the membranes were incubated with AIP56 that was denatured and diluted to a final concentration of 0.06 M GuHCl. However, when the experiment was performed with denatured AIP56 diluted to 0.6 M GuHCl, a stronger, concentration-dependent binding to the immobilized cyclophilins was detected. Interestingly, although no binding of native AIP56 to immobilized Hsp90 was detected, a strong binding was observed when the membranes were incubated with denatured toxin (Fig. 3A). Taken together, these results indicate that AIP56 interacts with Hsp90 and cyclophilins A and D *in vitro*. The marked effect of AIP56 denaturation on the observed bindings suggests that the interaction occurs preferentially when the toxin is unfolded and is consistent with the hypothesis that Hsp90 and cyclophilins interact with AIP56 during or immediately after translocation, when the toxin likely is in an at least partially unfolded state.

### Hsp90 interacts with AIP56 during cell intoxication

Prompted by the results of the dot-blot assays showing that AIP56 interacts with Hsp90 and cyclophilins *in vitro*, we performed pull-down experiments to investigate if similar interactions are detected in lysates obtained from intoxicated cells. We have previously shown that intoxication by AIP56 is initiated by binding of the toxin to an unknown cell-surface receptor of the target cells, followed by receptor-mediated endocytosis^7^. After endocytosis, the toxin reaches early endosomes, where it remains for 20 to 30 min^7^ until translocation from this compartment into the cytosol through a mechanism dependent on endosome acidification and likely involving low pH-induced unfolding^7^. Taking this into consideration, and with the aim of investigating if AIP56 interacts with Hsp90 and cyclophilins during intoxication, we performed pull-down assays using cytosolic fractions obtained from mBMDM that were incubated with AIP56V5 for 30 or 45 min. Cytosolic fractions obtained from untreated cells were used as controls. Beads coated with an anti-AIP56 antibody were used to immunoprecipitate AIP56 and the co-precipitation of Hsp90 and cyclophilins A and D was investigated by western blotting. Input or unbound protein controls, taken from the respective samples prior to or after bead incubation, respectively, were used to assess protein loading. As shown in Fig. 3B, co-precipitation of Hsp90 with AIP56 was detected in cytosolic fractions obtained from cells incubated with the toxin for 30 min, but not in cytosols from cells incubated with AIP56 for 45 min, neither in samples from untreated cells. Co-precipitation of cyclophilin A and D was not detected in any of the conditions tested (not shown). It has been previously shown that in mBMDM, AIP56 translocation occurs after 15–30 min after incubation with the toxin^7^. Therefore, the observed kinetic of the AIP56/Hsp90 interaction is consistent with the hypothesis that Hsp90 facilitates AIP56 intoxication by assisting the translocation of the toxin into the cytosol or its refolding shortly after translocation.

### The interaction with Hsp90/cyclophilins involves the catalytic domain of AIP56

**Figure 4:**
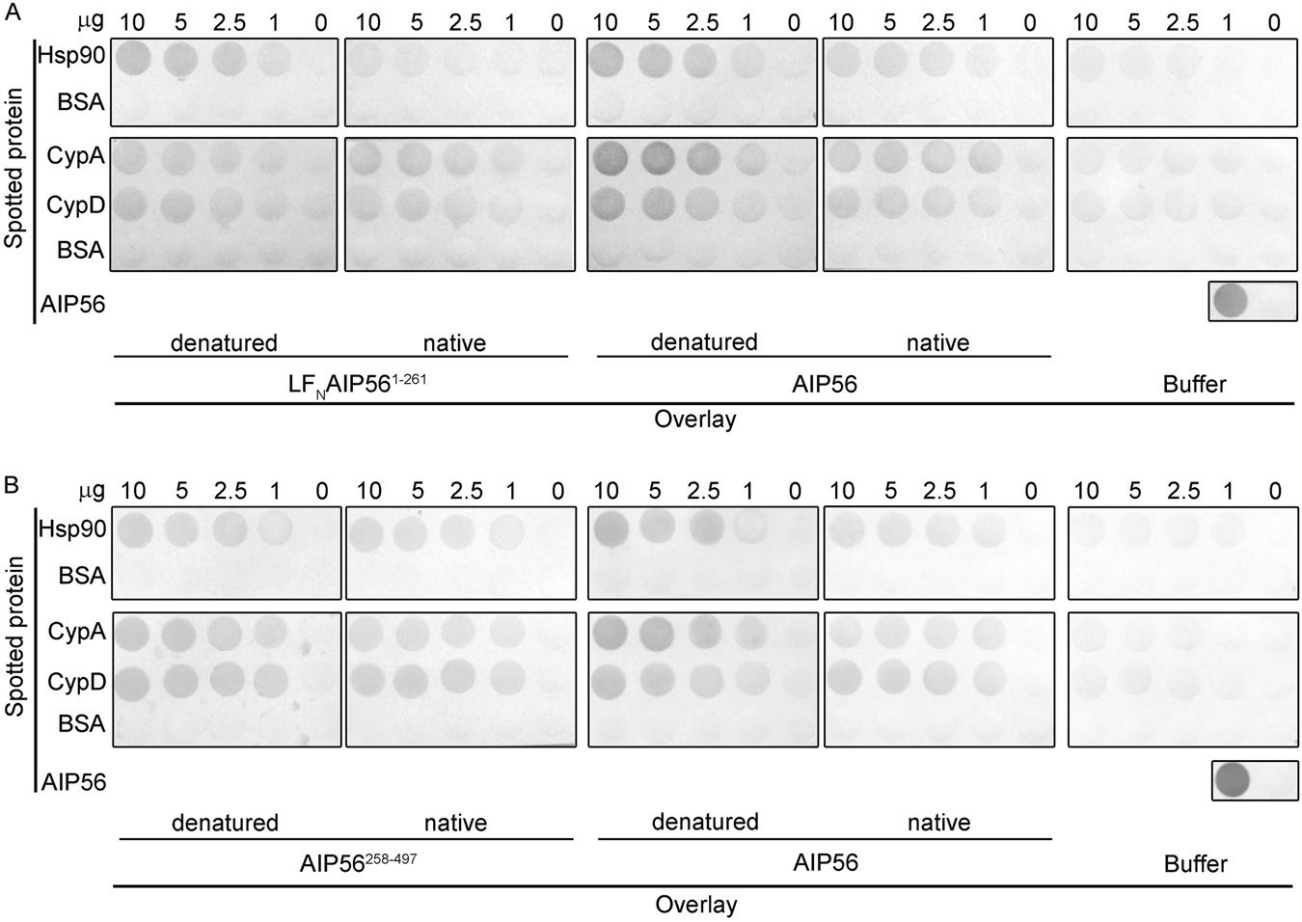
Dot blot analysis of the interaction between the catalytic and the transport domains of AIP56 with Hsp90, cyclophilin A and cyclophilin D. Membranes spotted with Hsp90, CypA, CypD, BSA (irrelevant protein) or AIP56 (positive control) were incubated with: (**A**) native LF_N_AIP56^1–261^ or AIP56, or with the same proteins previously denatured by incubation in 6 M GuHCl and diluted to a final concentration of 0.6 M GuHCl. As control, equivalent membranes were incubated with buffer without toxins. The interaction of LF_N_AIP56^1–261^ or AIP56 with the immobilized proteins was detected with an anti-AIP56^1–285^ antibody (chromogenic detection). (**B**) native AIP56^258–497^ or AIP56, or with the same proteins previously denatured by incubation in 6 M GuHCl and diluted to a final concentration of 0.6 M GuHCl. As control, equivalent membranes were incubated with buffer without toxins. The interaction of AIP56^258–497^ or AIP56 with the immobilized proteins was detected with an anti-AIP56^286–497^ antibody (chromogenic detection). Each blot was repeated at least three times. The blots shown in each panel were originally from the same membrane that was cropped to allow separate incubations with the different overlay mixtures. After these incubations and in all the subsequent steps, the spotted with Hsp90 or CypA/D were grouped and each group processed together in the same container. Ponceau S stainings of the membranes are shown in Fig. S6.

It has been shown that during the translocation process of several ADP-ribosylating toxins that require Hsp90 and PPIases for efficient intoxication, their catalytic domains are involved in the direct interaction with those host cell helpers (reviewed by^22^). Prompted by these findings, we directed our investigation to understand which regions of AIP56 were involved in the interaction with Hsp90 and cyclophilins. We started by performing dot-blot experiments to investigate if the AIP56 catalytic or transport domains were able to interact with Hsp90, CypA and CypD *in vitro*. In these experiments, we used LF_N_AIP56^1–261^ or AIP56^258–497^ as the overlay proteins and assessed binding under native and denatured conditions (Fig. 4). For comparative purposes, full-length AIP56 was always included as overlay protein in the dot blots with LF_N_AIP56^1–261^ or AIP56^258–497^.

As shown in Fig. 4, both LF_N_AIP56^1–261^ and AIP56^258–497^ bound to CypA and CypD, although the signals obtained were weaker that the one observed with the full-length toxin, suggesting that both the catalytic and transport domains contribute to the binding observed *in vitro* with the full-length toxin. Furthermore, contrary to what was observed with full-length AIP56, binding of the individual domains to CypA and CypD was not affected by the folding state of the overlay proteins. This suggests that the binding regions in the isolated domains are already exposed under native conditions, whereas in the full-length toxin their exposure is increased when the toxin is unfolded.

Concerning the interactions of the isolated domains with Hsp90 *in vitro*, the results of the dot-blots show that the catalytic domain of the toxin displays a binding pattern similar to the full-length toxin, with increased binding under denatured conditions (Fig. 4A), whereas the binding of the transport domain is weaker and does not increase when the overlay protein is unfolded (Fig. 4B). Together, the results obtained in the dot-blots suggest that the catalytic domain of AIP56 is the main responsible for the binding to Hsp90 observed *in vitro* with the full-length toxin under denatured conditions.

**Figure 5:**
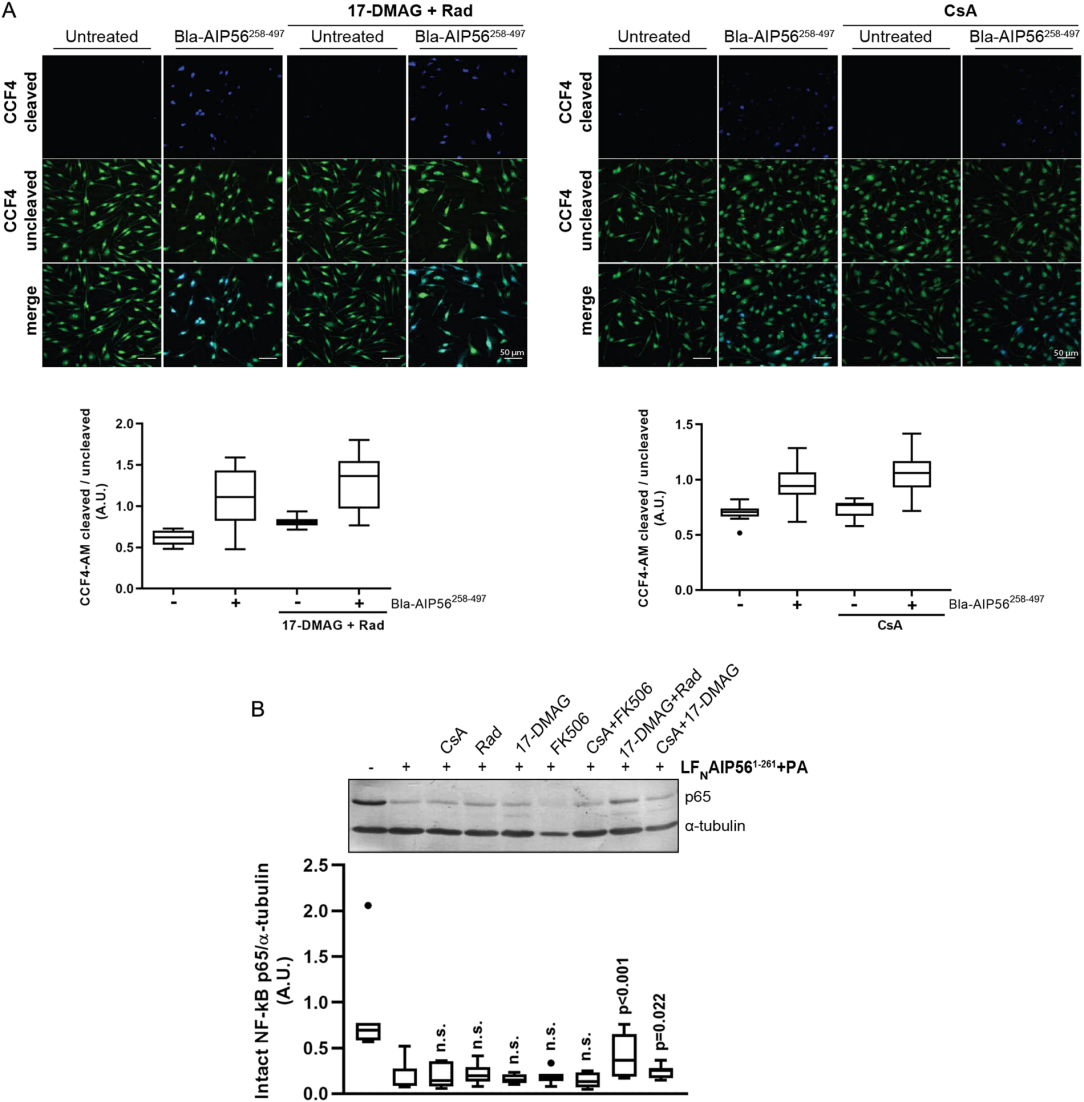
The assistance of AIP56 intoxication by Hsp90 and cyclophilins involves the catalytic domain of AIP56. (**A**) Pharmacological inhibition of Hsp90 and cyclophilins do not affect the cellular uptake of Bla mediated by the AIP56 B domain. mBMDM were pre-treated with 17-DMAG + Rad or CsA, incubated Bla-AIP56^258–497^ for 15 min at 37 °C and loaded with CCF4-AM for 30 min. Untreated cells and cells treated only with the inhibitors were used as controls. Uncleaved and cleaved CCF4-AM were detected by microscopy and the ratios determined by quantifying a minimum of 10 microscopic fields for condition. Results shown represent one out of three independent experiments. Statistical significance between mean increments in the ratio of cleaved/uncleaved CCF4-AM induced by Bla-AIP56^258–497^ in the presence or absence of the drugs was tested by the t-test and Mann-Whitney test (n = 3 independent experiments) and no difference was observed. (**B**) Cellular uptake of LF_N_AIP56^1–261^ is prevented by pharmacological inhibition of Hsp90. mBMDM were pre-treated with Rad, 17-DMAG, CsA or FK506, alone or combined, and intoxicated with LF_N_AIP56^1–261^ + PA in the presence of the inhibitor(s). Cleavage of p65 was detected by western blotting (chromogenic detection). A representative blot is shown (the full blot is presented in Fig. S7). The box-plot shows the quantification of the blots (n = 6 independent experiments). Loading correction was achieved by dividing the density of p65 by the respective density of α-tubulin. Statistical significance was tested by one-way ANOVA. p-values for individual comparisons were calculated using the Dunnett’s test and refer to comparisons to cells treated only with toxin.

After testing the binding of the AIP56 A and B domains to the chaperone *in vitro*, we performed intoxication assays to determine which of the observed interactions may be relevant during cell intoxication (Fig. 5). The biologic relevance of the interactions detected *in vitro* with the AIP56 transport domain was investigated using a beta-lactamase (Bla)-AIP56^258–497^ chimera, in which the catalytic domain of AIP56 was replaced by Bla^19–286^. This chimera was incubated with mBMDM, and the arrival of the Bla moiety at the cytosol was evaluated by a FRET-based assay using the CCF4-AM substrate^33^. This fluorescent substrate consists of a cephalosporin backbone that connects coumarin and fluorescein molecules. Excitation of coumarin results in FRET to the fluorescein (Ex of 409 nm to Em of 520 nm), which emits a green fluorescence signal. In the presence of Bla, this linker is cleaved leading to FRET disruption and a blue-shifted fluorescence (Ex of 409 nm to Em of 447 nm). Intact and cleaved substrates in mBMDM were detected by microscopy and quantified using the Fiji software. In order to normalize substrate loading, the results were expressed as the ratio of cleaved to intact CCF4-AM, as described^34^. As shown in Fig. 5A, incubation of mBMDM with Bla-AIP56^258–497^ resulted in the cleavage of CCF4-AM, indicating that the Bla moiety reached the cytosol of the cells. The cleavage of CCF4-AM was not affected by pre-treatment with CsA or 17-DMAG + Rad (Fig. 5A), indicating that although the AIP56 B domain is able to interact with the chaperones *in vitro*, the cellular entry of the Bla moiety mediated by the AIP56 B domain does not require the participation of Hsp90 and cyclophilins.

To investigate the involvement of the AIP56 catalytic domain in the interaction with Hsp90 and cyclophilins during intoxication, we used a protein chimera consisting of the AIP56 catalytic domain (AIP56^1–261^) fused to an N-terminal peptide of LF, previously shown to be efficiently delivered into the cytosol of mBMDM through the protective antigen (PA) pore^6^. It has been reported that the protective antigen (PA)-mediated cellular uptake of lethal factor (LF) from *Bacillus anthracis* is independent on Hsp90 and cyclophilins and does not involve interactions of the toxin with these host cell proteins^25^, which allowed the use of the LF/PA delivery system to study the involvement of the catalytic domains of ADP-ribolsylating toxins in the interaction with Hsp90 and PPIases during intoxication^25^. We also took advantage of this system, and performed intoxication assays with LF_N_AIP56^1–261^ + PA in mBMDM pre-treated with CsA, FK506, Rad or 17-DMAG, alone or combined, to assess if the inhibitors affected the AIP56-dependent p65 cleavage.

As shown in Fig. 5B, when Rad was applied to cells in combination with 17-DMAG, a significant inhibition of p65 cleavage was observed, suggesting that assistance of AIP56 intoxication by Hsp90 involves the toxin catalytic domain. In contrast to the effect of the pharmacological inhibition of Hsp90 with 17-DMAG + Rad, inhibition of cyclophilins with CsA did not affect p65 cleavage after treatment with LF_N_AIP56^1–261^ + PA (Fig. 5B), suggesting that the ability of the AIP56 catalytic domain to bind CypA and CypD *in vitro* is not biologically relevant during intoxication with LF_N_AIP56^1–261^.

Altogether, the results of the *in vitro* studies with the AIP56 isolated domains, and the intoxication assays performed and the LF_N_AIP56^1–261^ and Bla-AIP56^258–497^, do not allow drawing conclusions about the role of the metalloprotease domain of AIP56 in binding cyclophilins, but strongly suggest that this region is the one determining the dependence on Hsp90 for intoxication.

## Discussion

In the present study, we showed that Hsp90 and cyclophilin A/D are involved in assisting macrophage intoxication by the metalloprotease toxin AIP56.

It is known that host cytosolic factors, including host cell chaperones such as heat shock protein 90 (Hsp90) and peptidyl-prolyl *cis/trans* isomerases (PPIases), namely cyclophilin A/D (Cyp) and FK506-binding proteins (FKBP) are involved in the uptake of several bacterial AB toxins with ADP-ribosylating activity^14,18,19,21,23^, but are dispensable for the uptake of other AB toxins with different enzymatic activities, such as *Bacillus anthracis* lethal toxin (a metalloprotease)^24,25^ or the large glycosylating toxins A and B of *Clostridium difficile*^15,17–19^. The current view is that during translocation into the cytosol in an at least partially unfolded conformation, the ADP-ribosyltransferase domain of ADP-ribosylating toxins interact with a multiprotein complex that includes Hsp90/Cyp/FKBP that assists their translocation and refolding at the cytosol^22^. Although many details of binding of the Hsp90 with its clients remain poorly understood, it is accepted that Hsp90 has an extended substrate binding interface and exhibits specificity for proteins with hydrophobic residues spread over a large area^35^. Since our previous work showed that AIP56 is a short-trip toxin that translocates from endosomes into the cytosol through a mechanism requiring endosome acidification and involving low pH-induced conformational changes in the toxin molecule that leads to toxin unfolding and increased hydrophobicity^7^, we hypothesized that Hsp90 and possibly other folding helpers such as Cyp and FKBP could assist membrane translocation of AIP56 and/or its refolding after translocation.

To test this hypothesis, we evaluated the effect of the pharmacological inhibition of host cell´s Hsp90, Cyp or FKBP in intoxication of mBMDM by AIP56 and found that inhibition of Hsp90 strongly inhibited AIP56 toxicity. Inhibition of Cyp slightly, but reproducibly, reduced intoxication, whereas specific inhibition of FKBP had no effect on toxicity. These results implicate Hsp90 and PPIases of the Cyp family as cytosolic factors assisting AIP56 intoxication. The presence of the inhibitors did not affect the endocytic uptake of AIP56 neither interfered with its catalytic activity, strongly suggesting that the protection observed is likely associated with membrane translocation and/or refolding of AIP56 at the host cell cytosol. Results obtained in co-immunoprecipitation experiments support this interpretation, as Hsp90 co-precipitated with AIP56 in lysates from cells treated with the toxin for 30 min, a period consistent with the AIP56 translocation time^7^. It is worth to note that the Hsp90/AIP56 interaction here reported was observed in lysates prepared from intoxicated cells, i.e., in an approximate biological context.

It is widely known that Hsp90 serves as a platform for the folding and maturation of many client proteins^36^. For that, it interacts with a variety of co-chaperone proteins, assembling multi-chaperone complexes that fit the needs of specific client proteins and regulate the function of Hsp90^37^. These dynamic multi-chaperone complexes use the energy of ATP hydrolysis to drive conformational changes, thereby folding and activating their substrate proteins^38^. So far, although Hsp90 and CypA/D were found to be required for AIP56 translocation, it remains unclear whether these are part of a multiprotein complex, as attempts to detect a co-immunoprecipitated cytosolic complex of CypA/D and AIP56 were unsuccessful. However, the results obtained in dot blot experiments showed that Hsp90, CypA and CypD interacted directly with AIP56 *in vitro* and these interactions were stronger under denatured conditions. Our earlier studies showed that in response to the low pH found in endosomes, AIP56 undergoes reversible conformational changes that result in the exposure of hydrophobic residues, suggesting that similarly to other short-trip toxins, it translocates across the endosomal membrane in an unfolded state^7^. In line with that, finding that interaction of Hsp90 and CypA/D with AIP56 occurs preferentially when the toxin is unfolded, supports the hypothesis that Hsp90 and CypA/D may interact with AIP56 during or immediately after translocation to assist its refolding into an active conformation.

Several studies demonstrated that Hsp90 and Cyp assist translocation of ADP-ribosylating toxins by interacting directly and specifically with the toxin catalytic domain (reviewed in^39^). To investigate if assistance of AIP56 translocation by Hsp90 and Cyp also involved the metalloprotease domain of the toxin, we produced a chimeric protein consisting of the AIP56 catalytic domain fused to the N-terminal part of LF (LF_N_AIP56^1–261^), that has been previously shown to deliver the AIP56 catalytic domain into the cytosol of mBMDM through the protective antigen (PA) pore^6,7^. Intoxication assays showed that Hsp90 inhibitors protected the cells from toxicity by LF_N_AIP56^1–261^. Considering that PA-mediated uptake of LF is not dependent on Hsp90^25^, these results indicated that the catalytic moiety of AIP56 was involved in the interaction with Hsp90, although it could not be excluded that other regions of the wild type toxin were also mediating the assistance of intoxication by Hsp90. The involvement of the AIP56 enzymatic moiety in the interaction with Hsp90 during intoxication is also supported by the observation that the delivery of the Bla moiety at the cytosol of mBMDM after incubation with a fusion protein obtained by replacing the AIP56 catalytic domain by beta-lactamase (Bla-AIP56^258–497^) was not affected by inhibition of Hsp90. In agreement with this, dot blot assays performed with LF_N_AIP56^1–261^ showed that Hsp90 interacted directly with the AIP56 catalytic domain *in vitro*. The AIP56 catalytic domain also interacted with CypA and CypD *in vitro*, but the pharmacological inhibition of Cyp did not affect intoxication by LF_N_AIP56^1–261^. In face of this, it is not possible to conclude if the role of Cyp in assisting AIP56 intoxication involves interactions with the toxin’s metalloprotease domain. The here reported involvement of Hsp90 in AIP56 intoxication, together with the work of Tehran and colleagues showing that that Hsp90 is required for intoxication by clostridial neurotoxins, which are metalloproteases^40^, indicate that the assistance by Hsp90 is not restricted to ADP-ribosylating toxins. The same is true for Cyp, here shown to be required for intoxication by a toxin that does not harbor an ADP-ribosyltransferase domain. Based on these findings, we speculate that the participation of Hsp90 and Cyp in bacterial toxin entry may be more disseminated than initially expected, possibly including toxins with different catalytic activities. This is relevant, as it would open the door to the use of already developed pharmacological inhibitors of Hsp90 as well as novel CsA derivatives that are being developed for protecting from intoxication by ADP-ribosylating toxins^21^ for protection from intoxication by other toxins possessing different enzymatic domains.

## Methods

### Ethics statement

This study was carried out in accordance with European and Portuguese legislation for the use of animals for scientific purposes (Directive 2010/63/EU; Decreto-Lei 113/2013). The work was approved by Direcção-Geral de Alimentação e Veterinária (DGAV), the Portuguese authority for animal protection (ref. 004933).

### Reagents

Radicicol (R2146), cyclosporine A (C30024), FK506 (F4679) and PMSF (P7626) were purchased from Sigma Aldrich. 17-(Dimethylaminoethylamino)-17-demethoxygeldanamycin (17-DMAG; Cat. Code: ant-dgl-5) was purchased from Invivogen. HBSS, HEPES, L-glutamine and sodium pyruvate were purchased from Invitrogen. DMEM, fetal bovine serum (FBS) and penicillin/streptomycin (P/S) were fromGibco^TM^.

### Antibodies

The anti-cyclophilin A rabbit polyclonal antibody (ab41684) was purchased from Abcam. The anti-NF-kB p65 rabbit polyclonal antibody (sc-372), the anti-Hsp90 (sc-13119) and anti-GAPDH (sc-32233) mouse monoclonal antibodies were from Santa Cruz Biotechnology. The anti-sea bass NF-kB p65 rabbit serum was produced using a peptide located at the C-terminal region of sea bass p65 as antigen and previously characterised^6^. The anti-V5 (R960-25) and anti-tubulin (32–2500) mouse monoclonal antibodies were from Invitrogen. The anti-AIP56^1–285^ and anti-AIP56^286–497^ antibodies were produced at Davids Biotechnologie (Germany) by immunizing rabbits with recombinant AIP56^1–285C262S^ or AIP56^286–497C298S^, respectively Anti-AIP56^1–285^ and anti-AIP56^286–497^ purified IgG fractions were obtained from the immune sera by affinity purification using immobilised AIP56^1–285C262S^ or AIP56^286–497C298S^ as ligand. Goat anti-rabbit alkaline phosphatase (AF) conjugated secondary antibody (A9919) and goat anti-mouse AF conjugated secondary antibody (A2429) were from Sigma Aldrich. Sheep anti-rabbit horseradish peroxidase (HRP)-conjugated secondary antibody (AP311) and sheep anti-mouse HRP-conjugated secondary antibody (AP271) were from the Binding Site.

### Recombinant proteins

In this study, the following recombinant proteins were used**:** (i) AIP56 carrying a C-terminal His-tag (AIP56) (ii) AIP56 with a V5-tag plus His-tag at the C-terminus (AIP56V5); (iii) AIP56^258–497^ with a C-terminal His-tag (iv) LF_N_AIP56^1–261^ chimeric protein consisting on the amino-terminus of anthrax lethal factor (LF^11–263^) fused to AIP56 catalytic domain with a C-terminal His-tag; (v) protective antigen (PA) from *B. anthracis* (vi) Bla^19–286^AIP56^258–497^ chimeric protein consisting of the β-lactamase fused to the putative AIP56 B domain; (vii) mouse heat shock protein 90 alpha, class B member 1 (Hsp90ab1; NCBI accession number NP_032328) with His-tag at the C-terminus, (viii) mouse peptidyl-prolyl cis/trans isomerase A (cyclophilin A; NCBI accession number NP_032933) with His-tag at the C-terminus, (ix) mouse peptidyl-prolyl cis/trans isomerase D (cyclophilin D; NCBI accession number NP_080628) with His-tag at the C-terminus, (x) His-TccC3hvr toxin.

AIP56 and LF_N_AIP56^1–261^ were produced and purified as described in^6^ and AIP56V5 as described in^7^. PA was kindly provided by Steve Leppla (Microbial Pathogenesis Section, Laboratory of Parasitic Diseases, National Institute of Allergy and Infectious Diseases, Bethesda, USA). His-TccC3hvr toxin was kindly provided by Dr. Alexander E. Lang (Institute for Experimental and Clinical Pharmacology and Toxicology, Faculty of Medicine, University of Freiburg, Germany). Hsp90ab1, cyclophilin A and D were expressed in *E. coli* BL21-Star (DE3) strain (Invitrogen). Bacteria were grown at 37 °C in 1 L of LB medium supplemented with kanamycin at 100 μg ml^−1^ to an absorbance of 0.6–0.8 at 600 nm and protein expression was induced at 17 °C for Hsp90ab1 and at 25 °C for cyclophilin A and D for 20 h by adding 0.5 mM IPTG. Cells were harvested by centrifugation and resuspended in 40 ml of 50 mM Tris pH 8.0 with 300 mM NaCl. AIP56^258–497^ and Bla^19–286^AIP56^258–497^ were expressed in *E. coli* Rosetta (DE3) and *E. coli* BL21 (DE3) strains (Novagen), respectively, and induced at 17 °C. Cells were resuspended in 40 ml of 50 mM Bis-Tris pH 6.5 with 500 mM NaCl. In all cases, cell lysis was performed by sonication. After centrifugation, the recombinant proteins were purified from the supernatant using nickel-affinity chromatography (Ni-NTA agarose, Alfagene) followed by size exclusion chromatography (Superose 12 10/300 GL, GE Healthcare). The purity of the recombinant proteins (≥90%) was confirmed by SDS-PAGE.

### Determination of protein concentration

Concentrations of recombinant proteins were determined as previously described^6^ by measuring absorbance at 280 nm and using the extinction coefficient calculated by the ProtParam tool (http://www.expasy.org/tools/protparam.html), using the Edelhoch method^41^, but with the extinction coefficients for Trp and Tyr determined by Pace *et al*.^42^.

### Dot-blot assays

Different amounts of Hsp90, CypA and CypD diluted in 100 µl of 20 mM Tris-HCl pH 8.0, 200 mM NaCl were applied to a nitrocellulose membrane using a dot blot manifold. Vehicle or BSA (irrelevant protein) were used as negative controls and AIP56 was used as a positive control. The membrane was air dried and blocked for 30 min at room temperature (RT) with 5% skimmed milk in Tris-buffered saline (TBS) containing 0.1% Tween 20 (T-TBS). The membrane was then cut and incubated for 30 min at RT with: (i) 20 mM Tris-HCl, pH 8.0 + 200 mM NaCl; (ii) 170 nM AIP56, LF_N_AIP56^1–261^or AIP56^258–497^ in 20 mM Tris-HCl, pH 8.0 + 200 mM NaCl; (iii) AIP56, LF_N_AIP56^1–261^ or AIP56^258–497^ previously denatured by incubation in 6 M guanidine hydrochloride (GuHCl) for 1 h at RT and then diluted 10× or 100× in 20 mM Tris-HCl, pH 8.0 + 200 mM NaCl, to achieve a final concentration of 170 nM toxin, respectively, in 20 mM Tris-HCl, pH 8.0, 200 mM NaCl with 0.6 M or 0.06 M GuHCl, respectively. After 3 washes in T-TBS, the membrane was incubated for 1 h at RT with a rabbit polyclonal antibody against -AIP56^1–285^ or against AIP56^286–497^ or a mixture of both, diluted in blocking buffer (5% skimmed milk in T-TBS). Immunoreactive spots were detected using an alkaline phosphatase-conjugated secondary antibody followed by NBT/BCIP development.

### Experimental animals

The C57BL/6 mice were purchased from Charles River (Madrid, Spain) and bred and housed at the IBMC/i3S animal facility. The mice were fed sterilized food and water *ad libitum*. Mice were euthanized by isofluorane anesthesia followed by cervical dislocation.

### Cells

Mouse bone marrow derived macrophages (mBMDM) were derived from bone marrow of femurs and tibias from 4–12 week-old C57BL/6 male mice, as previously described^43^. Briefly, femurs and tibias were removed and flushed with 10 ml of Hanks’ balanced salt solution (HBSS). The resulting cell suspension was centrifuged and cells resuspended in supplemented DMEM (DMEM with 10 mM glutamine, 10 mM HEPES, 1 mM sodium pyruvate, 10% heat inactivated FBS, 1% penicillin/streptomycin and 10% of L929 cell conditioned medium (LCCM) as a source of Macrophage Colony Stimulating Factor (M-CSF)^44^. In order to remove fibroblasts, the cells were cultured overnight on a cell culture dish at 37 °C in a humidified chamber in a 7% CO_2_ atmosphere. The non-adherent cells were collected by centrifugation, resuspended in supplemented DMEM at a concentration of 5 × 10^5^ cells ml^−1^, plated in 24-well cell culture plates at a density of 5 × 10^5^ cells per well and incubated as above. Three days later, 10% LCCM (v/v) was added, at the 7^th^ day of culture the medium was renewed, and cells were used at day 10. To obtain LCCM, L929 cells were grown in 75 cm^2^ filtered cap flasks in supplemented DMEM until reaching 100% confluence. Cells were then diluted 1:100 in fresh supplemented DMEM and incubated for 10 days at 37 °C, 7.0% CO_2_. The supernatant was collect, pooled, centrifuged at 750 g for 10 min and filtered. LCCM was aliquoted and stored at −20 °C until used.

### Inhibition of toxicity in intact cells

Cells were pre-treated with cyclosporine A (20 µM), radicicol (10 µM), 17-DMAG (20 µM) and FK506 (20 µM) alone or combined in supplemented DMEM for 1 h at 37 °C prior to incubation with toxins. Stock solutions were prepared (3.33 mM cyclosporine and 5.48 mM radicicol in absolute ethanol, 8.1 mM 17-DMAG in water or 20 mM FK506 in DMSO) and diluted with culture medium on the day of the experiment to achieve the indicated final concentrations. Toxins were added to achieve final concentrations of 170 nM AIP56, 20 nM LF_N_AIP56^1–261^ + 10 nM PA or 50 ng ml^−1^ His-TccC3hvr + 100 ng ml^−1^ PA and incubated with the cells for 30 min on ice, followed by incubation for further 15 min at 37 °C. Cells were washed and incubated for further 2 h (AIP56) or 1 h (LF_N_AIP56^1–261^ + PA or His-TccC3hvr + PA) while maintaining the inhibitory conditions. Mock-treated cells, cells treated only with the toxins and cells treated only with inhibitors were used as controls. NF-κB p65 cleavage was evaluated by western blotting and was used as readout for intoxication by AIP56 and by LF_N_AIP56^1–261^ + PA^7^. Intoxication by His-TccC3hvr + PA was evaluated by analysing cell rounding by phase contrast microscopy, as described^20^.

### Detection of AIP56 proteolytic activity *in vitro*

The effect of the inhibitors in the catalytic activity of AIP56 *in vitro* was investigated using an *in vitro* catalytic assay, as described previously^6^. Briefly, sea bass peritoneal cell lysates were incubated for 3 h at 22 °C with 100 nM of recombinant AIP56 in a final volume of 20 µl of 10 mM Tris-HCl pH 8.0, 150 mM NaCl with or without inhibitors (20 µM CsA, 20 µM 17-DMAG, 10 µM Rad or 20 µM FK506, alone or combined). The reaction was stopped by adding SDS-PAGE sample buffer and p65 cleavage assessed by western blotting. Quantification of cleaved NF-kB p65 was performed by densitometry using Fiji software (ImageJ version 1.51n, NIH, USA). Loading correction was achieved by dividing the density of cleaved p65 by the respective density of Ponceau S.

### Detection of endocytosed AIP56

Endocytosis assays were performed by incubating mBMDM, seeded at a density of 1 × 10^5^ cells per well in a 96-well plate (Greiner 655090), with AIP56 labelled with Alexa Fluor 488 (AIP56-488) using the Alexa Fluor protein labelling kit (A-10235) from Molecular Probes, following the manufacturer’s instructions. The effect of the pharmacological inhibitors on endocytosis was accessed by pre-treating mBMDM with CsA (20 µM), Rad (10 µM), 17-DMAG (20 µM) or FK506 (20 µM), alone or in combination, in supplemented DMEM + 10% LCCM for 1 h at 37 °C. Cells treated with 80 µM of the dynamin inhibitor dynasore, previously shown to inhibit AIP56 endocytosis^7^, and untreated cells, were used as controls. The cells were then transferred to ice to acclimatize for 5 min, the medium removed and replaced by ice-cold medium with inhibitors plus 170 nM AIP56-488 and incubated for 30 min on ice followed by 15 min at 37 °C, as described above. After the incubations, the cells were fixed with 4% paraformaldehyde (PFA) in PBS for 15 min at RT and washed 3 times with ice cold PBS. Nuclei staining was performed using DAPI (5 µg ml^−1^) in PBS for 5 min at RT followed by PBS wash. The cells were kept in PBS protected from light until image acquisition using IN Cell Analyzer 2000 (GE Healthcare Life Sciences, USA) with Nikon 20 × /0.45 NA Plan Fluor objective and the DAPI and FITC filters. Untreated cells were used as control. Quantification of fluorescence intensity per cell was performed using Fiji software (ImageJ version 1.51n, NIH, USA). The integrated fluorescence intensity of the FITC channel was measured in each field of view (FOV) and the background subtracted. Number of cells per field was counted using ImageJ Cell Counter. In each experiment, a minimum of 500 cells per condition were analysed.

### Co-immunoprecipitation

All manipulations with cell lysates were carried out at 4 °C. Before each experiment, cells were incubated in a mixture of serum-free DMEM and HBSS (1:1) for 4 h and washed twice with PBS. Then, cells were incubated in serum-free medium with 285 nM AIP56 or without toxin (for control), for 15 min on ice followed by incubation for further 30 or 45 min at 37 °C. Cells were washed 3 times with PBS and resuspended in buffer (1 ml of lysis buffer per 6 × 10^5^ cells) containing 20 mM Tris-HCl pH 7.5, 150 mM NaCl, 1 mM EDTA, 1 mM EGTA, 1 µg ml^−1^ Leupeptin. Subsequently, cells were lysed by passage through a 25 G needle, centrifuged at 21,000 g for 15 min and supernatants collected. Protein A magnetic beads (Milipore, LSKMAGA10) (20 µL) and 2.5 µg anti-AIP56^1–285^ antibody were previously incubated in PBS for 1 h at RT with rotation. The cytoplasmic fraction from 6 × 10^6^ mBMDM was incubated with anti-AIP56^1–285^ antibody-protein A magnetic beads overnight at 4 °C with rotation. The beads were washed, heated for 10 min at 90 °C in SDS-PAGE sample buffer and the supernatants subjected to SDS-PAGE under reducing conditions. Immunoprecipitates were analysed by western blotting (ECL detection) for detection of Hsp90, cyclophilin A and cyclophilin D, as described below. Input or unbound fractions were run as controls. In all experiments, efficient intoxication of mBMDM by AIP56 was confirmed by assessing NF-κB p65 cleavage by western blotting.

### Fluorescence Resonance Energy Transfer (FRET) based assay

mBMDM cells cultured in 8-well plates (ibidi) were pre-treated with cyclosporine A (20 µM) or radicicol (10 µM) plus 17-DMAG (20 µM) in supplemented DMEM for 30 min at 37 °C. Next, the medium was replaced and Bla^19–286^AIP56^258–497^ was added at a final concentration of 525 nM in the presence of the inhibitors. The cells were incubated for 15 min at 37 °C, washed twice with PBS and loaded with CCF4-AM (Life Technologies; K1095), according to the manufacturer’s instructions, in Hanks’ balanced salt solution (HBSS) supplemented with 10 mM glutamine, 10 mM HEPES, 1 mM sodium pyruvate and 10% heat inactivated FBS for 30 min at RT. Subsequently, mBMDM were washed twice with supplemented HBSS and fixed at RT for 15 min in 4% (wt/vol) paraformaldehyde in Dulbecco’s Phosphate Buffered Saline (DPBS). Cells were then observed with CFI PL APO LAMBDA 40X/0.95 objective on a Nikon Eclipse Ti-E microscope (Nikon). The samples were illuminated with LED at 395 nm by a SpectraX light engine (Lumencor) using a quad dichroic filter 310DA/FI/TR/CY5-A and emission filters 450/50 and 525/50 (Semrock). Images were acquired with a EMCCD camera iXon ULTRA 888 (Andor Technologies). A minimum of 10 microscopic fields were analysed per condition in each experiment and 3 independent experiments were performed. Ratiometric analysis of images acquired for emissions wavelength (Em) 447 nm and 520 nm, corresponding to the ratio of cleaved CCF4-AM/intact CCF4-AM, was made by using a custom made ImageJ macro on Fiji software^45^. For each experiment, the increment in the ratio of cleaved/uncleaved CCF4-AM induced by Bla-AIP56^258–497^ incubation in the presence or absence of the drugs, was calculated as follows: increment = (ratio in Bla-AIP56^258–497^ treated cells – ratio in untreated cells)/ratio in untreated cells. Mean increments in the presence of the drugs were compared to mean increments in the absence of the drugs using the t-test and Mann-Whitney test (n = 3 independent experiments).

### SDS-PAGE and western blotting

SDS-PAGE was performed as previously described^7^, using the Laemmli discontinuous buffer system^46^. Before loading, samples were boiled for 5 min in SDS-PAGE sample buffer (50 mM Tris-HCl pH 8.8, 2% SDS, 0.05% bromophenol blue, 10% glycerol, 2 mM EDTA and 100 mM DTT). For western blotting analysis, the proteins were transferred onto nitrocellulose membranes. The efficacy of transfer and the protein loading on the membranes was checked by Ponceau S staining. The membranes were blocked for 1 h at RT with 5% skimmed milk in T-TBS followed by incubation for 1 h at RT with the primary antibodies diluted in blocking buffer. Immunoreactive bands were detected using alkaline phosphatase conjugated secondary antibodies and NBT/BCIP (Promega) or horseradish peroxidase-linked secondary antibodies and enhanced chemiluminescence (ECL) West Dura super signal substrate (Pierce Biotechnology). Blots shown correspond to representative results of at least 3 independent experiments. The quantification of the blots was performed by densitometry analysis using Fiji software. The results are expressed as the density of p65 band relative to the density of alpha-tubulin, GAPDH or Ponceau S staining, as indicated.

### Statistical analysis

Statistical analysis was performed using the IBM SPSS Statistics (v25) software. Original data is presented in box-plots. In each legend, the size of the experiment is indicated. Normality of the data was assessed through the Shapiro-Wilk test. In the case of the experiments involving quantification of NF-kB p65 cleavage (Figs 1, 2C, 5B and S3, S4) and quantification of endocytosis (Fig. 2B), data was log transformed so that normality was observed. In the experiment of Fig. S3, two extreme observations were discarded, resulting in a normal distributed data. In these experiments, the different incubation conditions were treated as a factor and the data obtained in each independent experiment as a block; therefore, the data was analysed considering a Randomized Block Design. One-tailed Dunnett pairwise test was used in Figs 1, 5B and S2 and the two-tailed Dunnett test was used in Figs 2B and S3, S4. Regarding the data in Fig. 5A, the mean increments in the ratio of cleaved/uncleaved CCF4-AM upon incubation with Bla-AIP56^258–497^ in the presence or absence of the drugs were compared using the t-test and Mann-Whitney test. Statistical significance was set for p < 0.05.

## Supplementary information

Supplementary Information

## Data Availability

All data and constructs are available upon request.
