# Author Correction: Involvement of Hsp90 and cyclophilins in intoxication by AIP56, a metalloprotease toxin from *Photobacterium damselae* subsp. *piscicida*

**DOI:** 10.1038/s41598-020-58152-x

**Published:** 2020-01-22

**Authors:** Inês S. Rodrigues, Liliana M. G. pereira, Johnny Lisboa, Cassilda Pereira, Pedro Oliveira, Nuno M. S. dos Santos, Ana do Vale

**Affiliations:** 1 Fish Immunology and Vaccinology Group, IBMC-Instituto de Biologia Molecular e Celular, Universidade do Porto, Porto, Portugal; 2 i3S-Instituto de Investigação e Inovação em Saúde, Universidade do Porto, Porto, Portugal; 3 EPIUnit, ICBAS-Instituto de Ciências Biomédicas Abel Salazar, Universidade do Porto, Porto, Portugal

Correction to: *Scientific Reports* 10.1038/s41598-019-45240-w, published online 21 June 2019

This Article contains an error in Figure 5A, where the incorrect image is shown for the CCF4 uncleaved channel of CsA/Untreated. The correct Figure 5 appears below as Figure 1.

**Figure 1:**
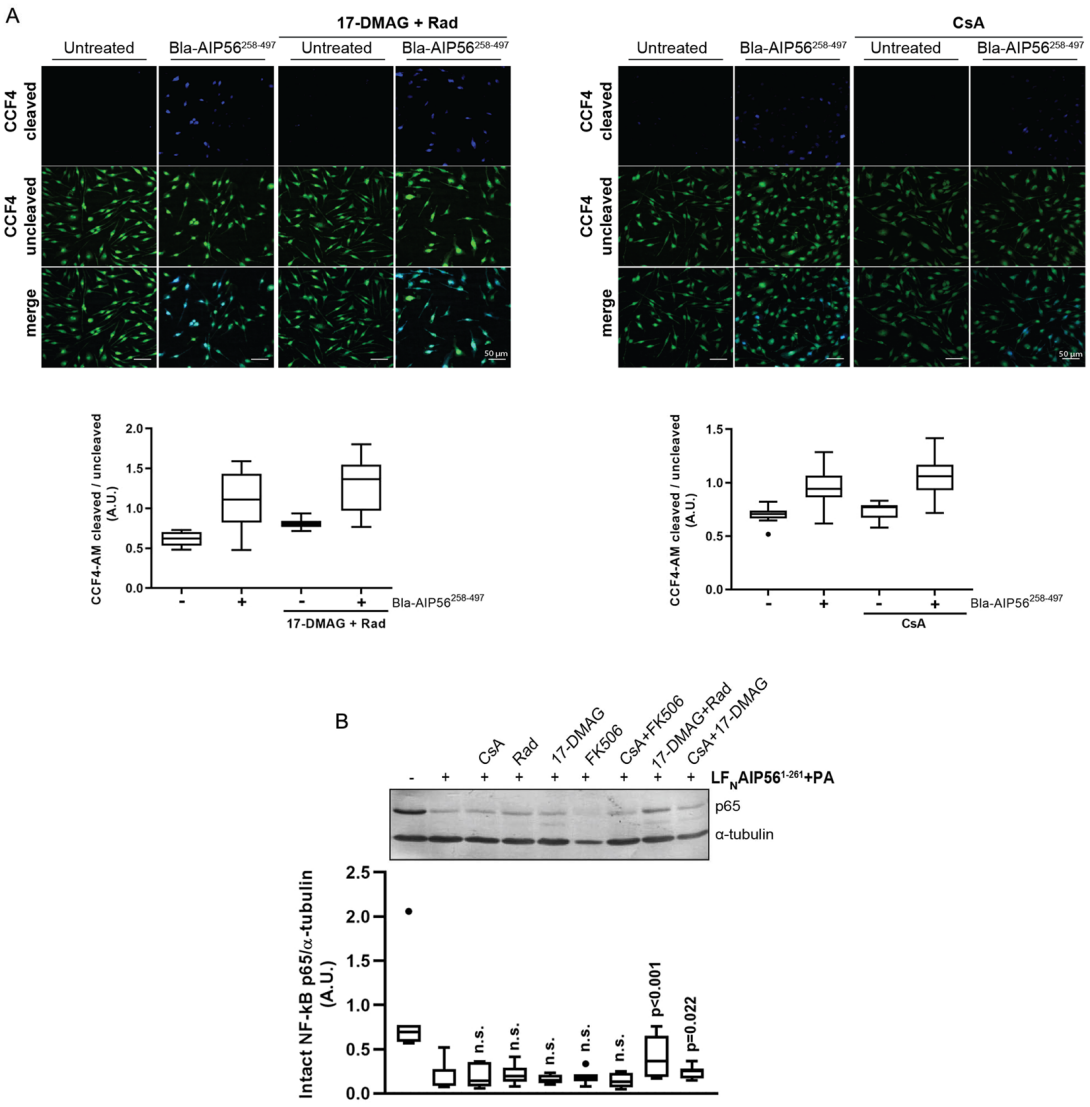
.

The conclusions of the Article are unaffected by these changes.

