# Supplementary material for: Involvement of Hsp90 and cyclophilins in intoxication by AIP56, a metalloprotease toxin from Photobacterium damselae subsp. piscicida
Source: Sci Rep. 2019 Jun 21;9:9019. doi: 10.1038/s41598-019-45240-w (PMC6588550; doi:10.1038/s41598-019-45240-w)
Supplement: Supplementary file 1 — Supplementary Information [file 41598_2019_45240_MOESM1_ESM.docx]

**Involvement of Hsp90 and cyclophilins in intoxication by AIP56, a metalloprotease toxin from *Photobacterium damselae* subsp. *piscicida***

Inês S. Rodrigues^1,2^, Liliana M. G. Pereira^1,2^, Johnny Lisboa^1,2^, Cassilda Pereira^1,2^, Pedro Oliveira^3^, Nuno M. S. dos Santos^1,2^, Ana do Vale^1,2^

**Supplementary information**

**
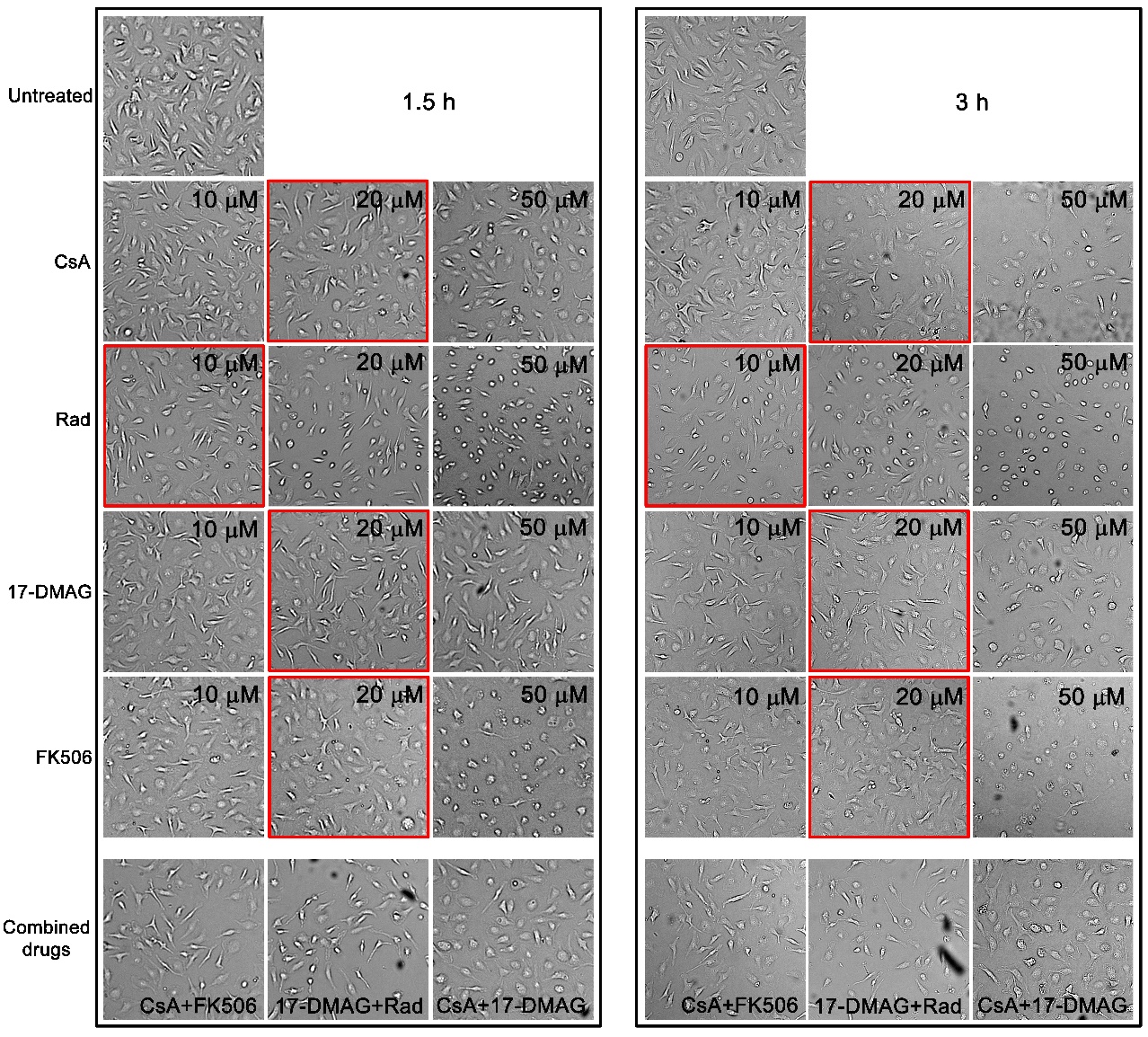
**

**Figure S1. Assessment of the toxicity of CsA, Rad, FK506 and 17-DMAG for mBMDM.** Mouse bone marrow derived macrophages (mBMDM) were left untreated or treated with 10, 20 or 50 µM of CsA, Rad, FK506 or 17-DMAG, or with the indicated inhibitor combinations (20 µM CsA + 20 µM FK506, 20 µM 17-DMAG + 10 µM Rad, 20 µM CsA + 20 µM 17-DMAG). Monolayers were observed by brightfield microscopy after 1.5 and 3 h to determine the maximum concentration of each inhibitor that was well tolerated by the cells (boxed in red). The experiment was repeated twice and the results shown are representative microscopic fields from one experiment. Images were acquired in an IN Cell Analyzer 2000 (GE Healthcare), a computer-controlled high-throughput widefield microscope using a Nikon 20X/0.45 NA Plan Fluor objective. The concentrations highlighted by red boxes and the inhibitor combinations shown were selected for subsequent experiments.

**

**

**Figure S2. Influence of CsA, Rad, FK506 and 17-DMAG on the toxicity of *Photorhabdus luminescens* ADP-ribosyltransferase TccC3**. Mouse bone marrow derived macrophages (mBMDM) were pretreated with the inhibitors (10 µM Rad and 20 µM 17-DMAG, CspA or FK506) for 1 h and were subsequently left untreated or intoxicated with 100 ng/ml PA + 50 ng/ml TccC3. (A) Phase contrast microscopy. Pictures were taken after 1 h incubation with the toxin (scale bar, 50 µm). (B) and (C) Quantitative analysis of cell rounding. In each experiment, three pictures from each condition were acquired and the percentage of cell rounding was determined. Box plots combine the results of three independent experiments. (B) No significant increase in cell rounding was observed after incubation of the cells with the drugs only, when compared to untreated cells. Statistical significance was tested by one-way ANOVA. p-values for individual comparisons were calculated using the Dunnett's test. (C) Pretreatment of cells with Rad, 17-DMAG, CsA or FK506 significantly inhibited intoxication by PA+His-TccC3. Statistical significance was tested by one-way ANOVA. p-values for individual comparisons were calculated using the Dunnett's test and refer to comparisons between groups pre-treated with the indicated drug followed by PA+His-TccC3 and the group treated only with PA+ His-TccC3.





**Figure S3. The presence of the inhibitors does not interfere with the NF-**κB **p65 level of mBMDM.** Mouse bone marrow derived macrophages (mBMDM) were pre-treated with 10 µM Rad, 20 µM 17-DMAG, 20 µM CsA or 20 µM FK506 for 1 h at 37 ºC. The medium was replaced with fresh medium with inhibitor(s) and cells incubated for further 2 h at 37 ºC. The levels of p65 was determined by western blotting (chromogenic detection). A representative blot is shown (the full blot is presented in Fig. S7). The box plot shows the quantification of the blots (n=3 independent experiments). Loading correction was achieved by dividing the density of p65 by the respective density of α-tubulin. Statistical significance was tested by one-way ANOVA. p-values for individual comparisons were calculated using the Dunnett's test and refer to comparisons to untreated cells.


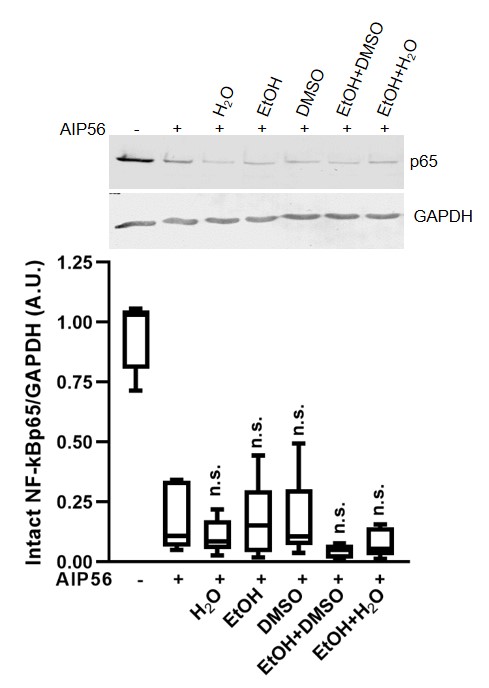


**Figure S4. The vehicles used to dissolve the inhibitors do not inhibit AIP56-dependent NF-κB p65 cleavage.** Mouse bone marrow derived macrophages (mBMDM) were left untreated or pre-treated with the different vehicles/vehicle combinations (H_2_O, vehicle for 17-DMAG; EtOH, vehicle for CsA and Rad; DMSO, vehicle for FK506) for 1 h at 37ºC prior to incubation with 170 nM AIP56 for 30 min on ice plus 15 min at 37ºC. Medium was replaced by fresh medium with the vehicles/vehicle combinations, and after 1.5 h at 37 ºC, cleavage of p65 was accessed by western blotting (chromogenic detection). A representative blot is shown (the full-length blot is shown in Fig. S7). The box-plot shows the quantification of the blots (n=5 independent experiments). Loading correction was achieved by dividing the density of p65 by the respective density of GAPDH. Statistical significance was tested by one-way ANOVA and p-values for individual comparisons to cells treated only with AIP56 were calculated using the Dunnett’s test.


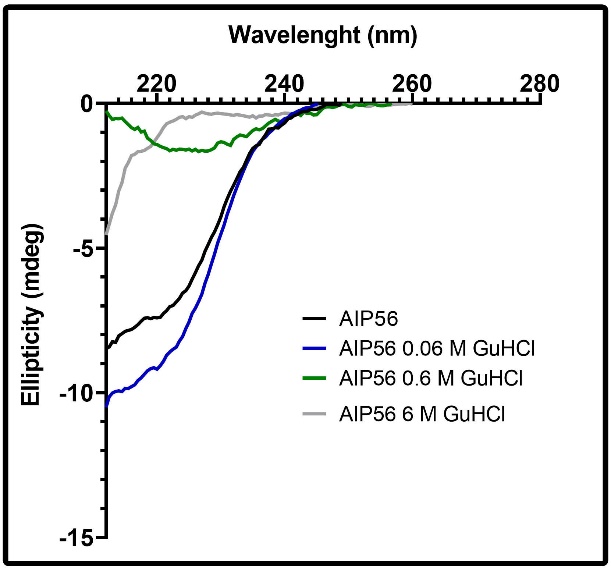


**Figure S5. GuHCl-induced unfolding of AIP56 monitored by CD.** CD spectra of 0.1 mg/ml AIP56 in the presence of 0.06 M, 0.6 M and 6 M GuHCl. Unfolding of AIP56 induced with 0.6 M and 6 M of GuHCl revealed a strong decrease in the ellipticity below 240 nm. The most dramatic change occurred at 215-230 nm, where the pronounced CD signatures of native AIP56, observed both in AIP56 and AIP56 with 0.06 M GuHCl, are lost when AIP56 is in the presence of 0.6 M and 6 M GuHCl. CD spectra were recorded at 20 ºC in a 1 mm path-length cuvette, from 260 to 190 nm with a scanning speed of 50 nm/min, a spectra bandwidth of 1 nm, and a integration time of 2 s. Data shown is the average of 16 scans after blank subtraction.

**Figure S6. Ponceau S staining of the dot blots of Fig. 3A, 4A and 4B.**

**Fig. 3A:**

**
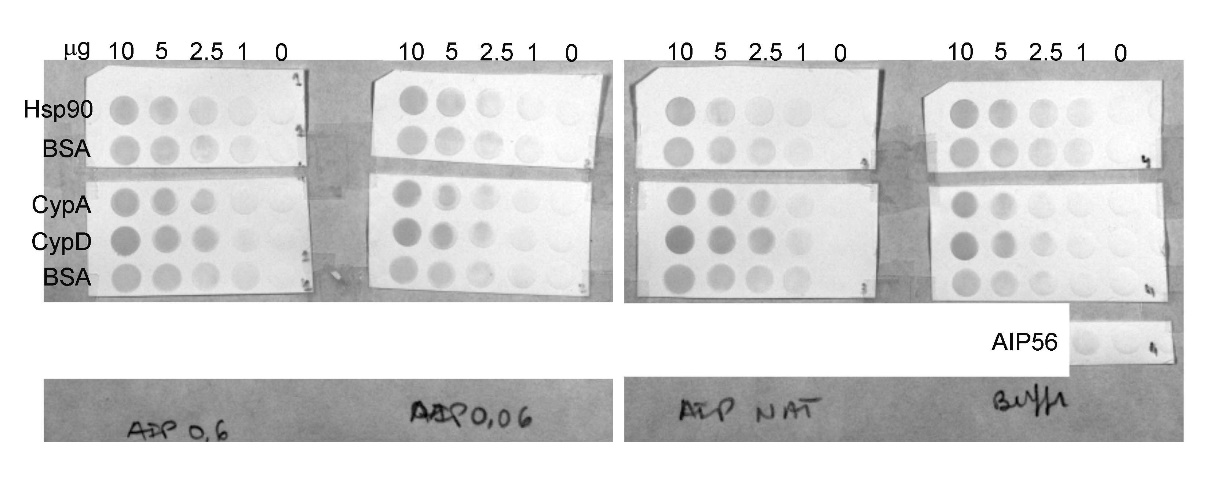
**

**Fig.4A:**

**
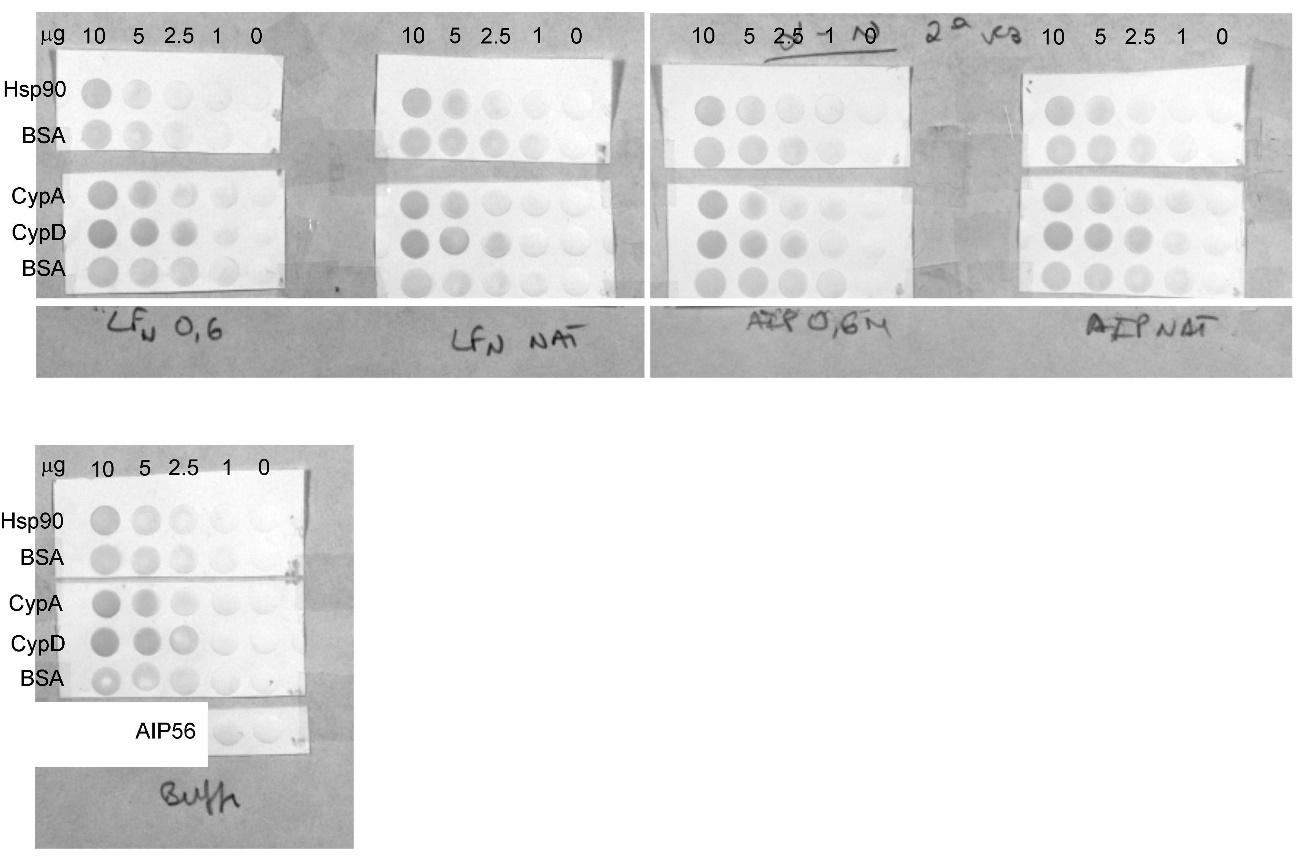
**

**Fig.4B:
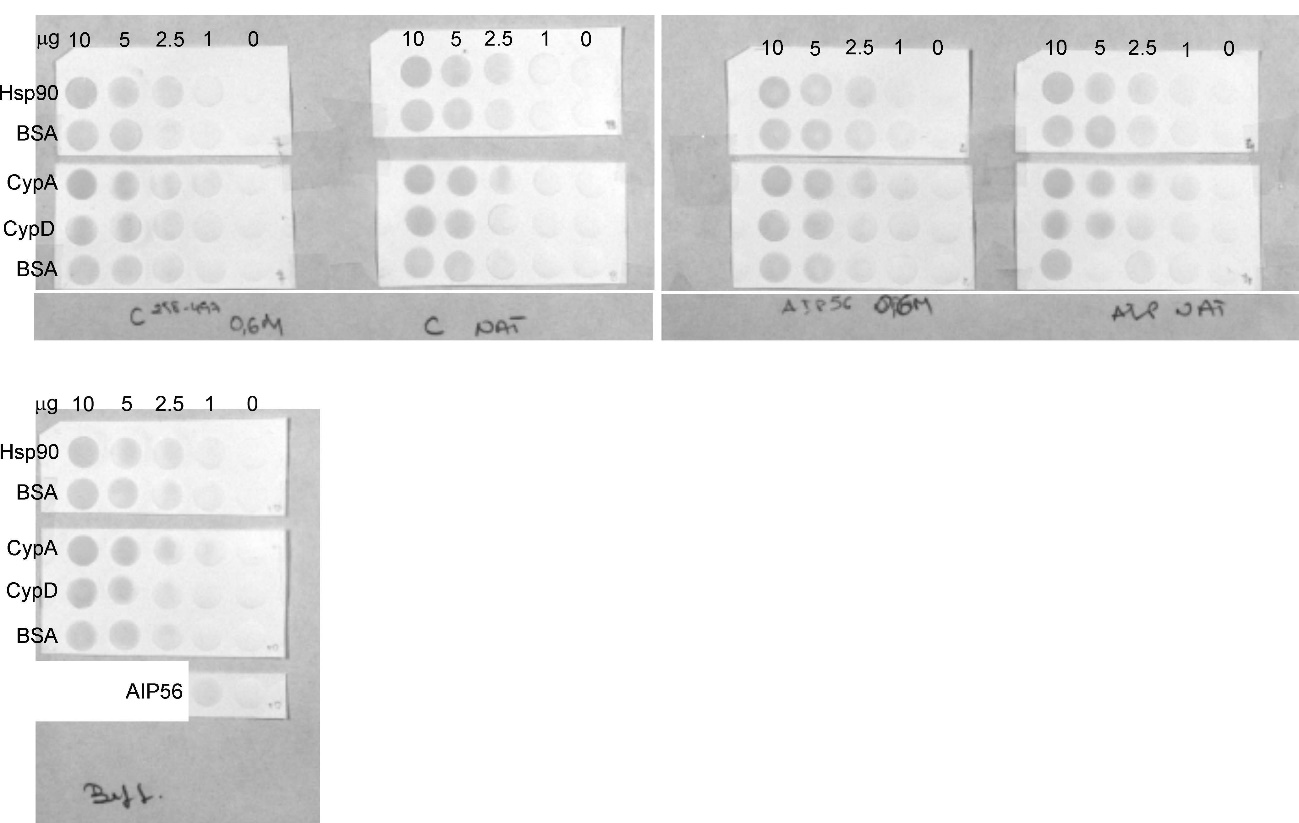
**

**Figure S7. Full blots of Fig. 1, 2C, 3B, 5B, S3 and S4.** Areas presented in the manuscript figures are boxed in red.

**Fig.1:**

**Fig. 2C:**

**
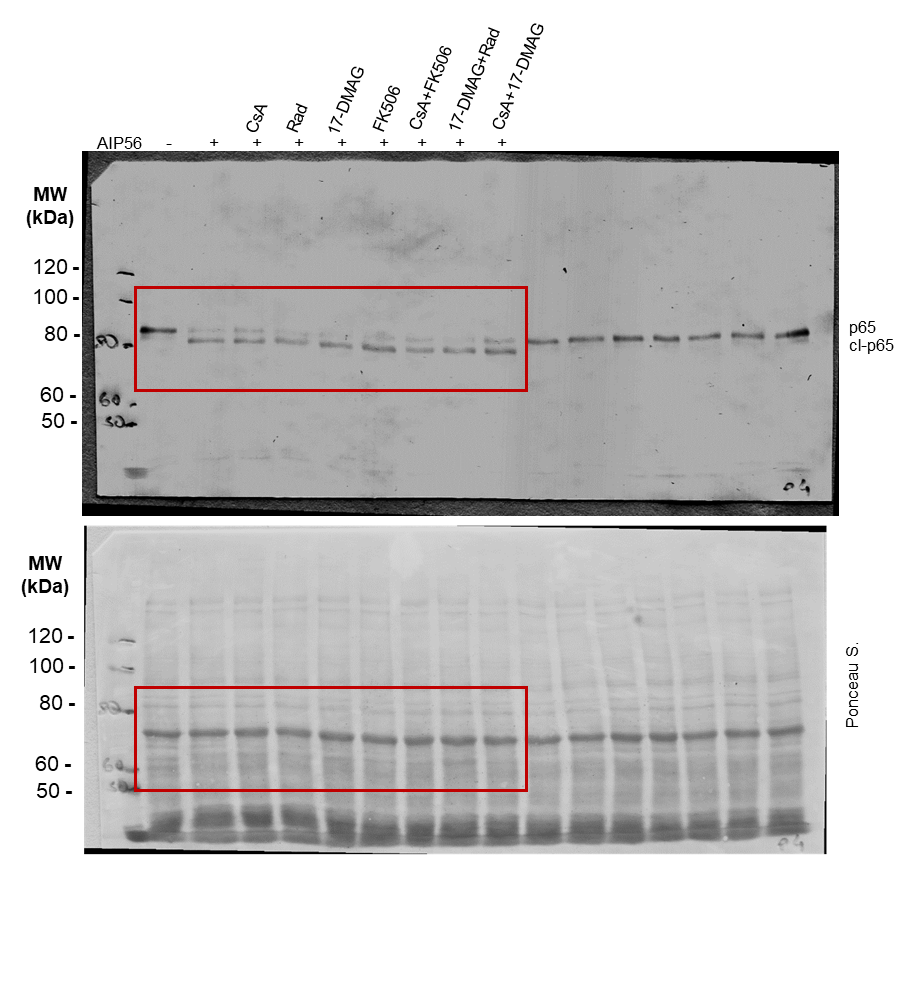
**

**Fig. 3B:**

**Fig. 5B:**

**Fig. S3:**

**Fig. S4:**

**
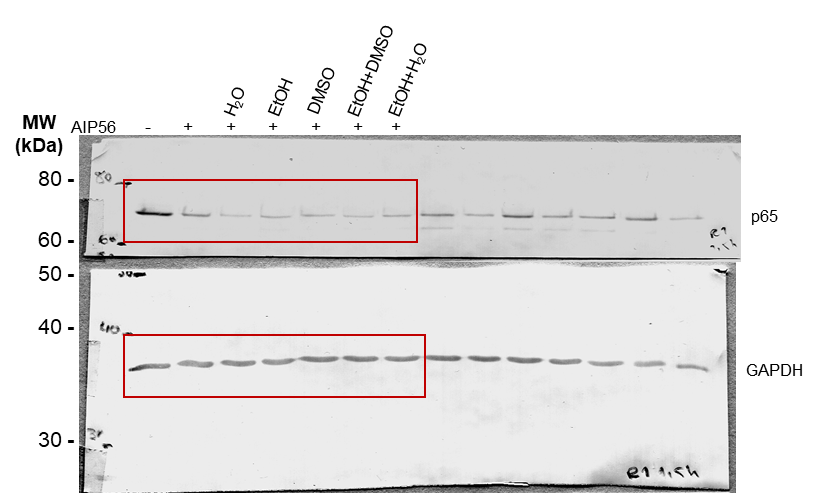
**
